# *UDP-GLYCOSYLTRANSFERASE 72E3* Plays a Role in Lignification of Secondary Cell Walls in *Arabidopsis*

**DOI:** 10.3390/ijms21176094

**Published:** 2020-08-24

**Authors:** Fabien Baldacci-Cresp, Julien Le Roy, Brigitte Huss, Cédric Lion, Anne Créach, Corentin Spriet, Ludovic Duponchel, Christophe Biot, Marie Baucher, Simon Hawkins, Godfrey Neutelings

**Affiliations:** 1 University Lille, CNRS, UMR 8576, Unité de Glycobiologie Structurale et Fonctionnelle (UGSF), F-59000 Lille, France; fabien.baldacci-cresp@univ-lille1.fr (F.B.-C.); JLeroy@lacitec.on.ca (J.L.R.); brigitte.huss@univ-lille.fr (B.H.); cedric.lion@univ-lille.fr (C.L.); anne.creach@univ-lille.fr (A.C.); corentin.spriet@univ-lille.fr (C.S.); christophe.biot@univ-lille.fr (C.B.); simon.hawkins@univ-lille.fr (S.H.); 2 College La Cité, Technology Access Center in Bio-Innovation (TAC-B), Ottawa, ON K1K4R3, Canada; 3 University Lille, CNRS, Inserm, CHU Lille, Institut Pasteur de Lille, US 41, UMS 2014, PLBS, F-59000 Lille, France; 4 University Lille, CNRS, UMR 8516, LASIR, Infrared and Raman Spectroscopy Laboratory, F-59000 Lille, France; ludovic.duponchel@univ-lille.fr; 5 Laboratoire de Biotechnologie Végétale (LBV), Université libre de Bruxelles, B-6041 Gosselies, Belgium; mbaucher@ulb.ac.be

**Keywords:** lignin, UDP glycosyltransferase, glycosylation, monolignol incorporation, cell wall

## Abstract

Lignin is present in plant secondary cell walls and is among the most abundant biological polymers on Earth. In this work we investigated the potential role of the *UGT72E* gene family in regulating lignification in *Arabidopsis*. Chemical determination of floral stem lignin contents in *ugt72e1*, *ugt72e2,* and *ugt72e3* mutants revealed no significant differences compared to WT plants. In contrast, the use of a novel safranin O ratiometric imaging technique indicated a significant increase in the cell wall lignin content of both interfascicular fibers and xylem from young regions of *ugt72e3* mutant floral stems. These results were globally confirmed in interfascicular fibers by Raman microspectroscopy. Subsequent investigation using a bioorthogonal triple labelling strategy suggested that the augmentation in lignification was associated with an increased capacity of mutant cell walls to incorporate H-, G-, and S-monolignol reporters. Expression analysis showed that this increase was associated with an up-regulation of *LAC17* and *PRX71*, which play a key role in lignin polymerization. Altogether, these results suggest that UGT72E3 can influence the kinetics of lignin deposition by regulating monolignol flow to the cell wall as well as the potential of this compartment to incorporate monomers into the growing lignin polymer.

## 1. Introduction

Lignin is a major structural component of many plant cell walls. The appearance of this polymer is associated with terrestrialization since it is responsible for the mechanical support, essential for upright growth, but also for providing a hydrophobic environment to the cells specialized in the conduction of water [1]. As well as playing a central role in the biology of vascular plants, lignin also has a major impact on the quality of a wide range of economically important products derived from plant lignocellulosic biomass [2]. The composition of the lignin polymer can be very complex but the major constituents derive from the oxidative coupling of three *p*-hydroxycinnamyl alcohols (monolignols), *p*-coumaryl, coniferyl and sinapyl alcohols, differing in their degree of methoxylation. After incorporation into the lignin polymer, these monomers give rise to *p*-hydroxyphenyl (H), guaiacyl (G), and syringyl (S) units respectively [3]. Beyond these traditional monolignols, the polymer can also include other phenylpropanoids [4,5] including caffeyl alcohol in specific cactus and orchid species [6,7], tricin flavonoids and ferulates in grasses [8,9] or hydroxystilbenes in palm fruit [10].

Given [11] the importance of the lignin polymer for both the plant and for the economy, scientists have long investigated how plants synthesize monolignols and regulate lignin levels in the cell wall. The monolignol biosynthesis pathway starts with phenylalanine and/or tyrosine (derived from the shikimate pathway) that are used as initial substrates [1]. These are then processed through a series of enzymatic activities leading to the deamination, reduction, hydroxylation, and *O*-methylation of the intermediates in the cytoplasm [1,12]. The genes encoding the corresponding enzymes are mostly part of multigenic families with variable sizes and whose members may have redundant functions or not. In addition to their role in monolignol production, different enzymes can be specialized in the production of many different phenylpropanoids including flavonoids, stilbenes, coumarins, phenylpropenes, or hydroxycinnamic acids [13]. Experiments have shown that both lignin levels and chemical composition are modified by engineered changes in the expression of different genes involved in monolignol biosynthesis leading to the redirection of the carbon flux into another biosynthetically related compound or group of compounds (reviewed in [14]). 

Following monolignol biosynthesis, the different lignin precursors are exported to the cell wall by mechanisms that still remain unclear. To some extent, ATP-binding cassette (ABC) transporter families seem to be involved in the transport through the plasma membrane. This was suggested by the demonstration that *p*-coumaryl alcohol membrane transport was induced via the heterologous expression of *ABCG29* in yeast [15], as well as by the observation that several ABCG genes are co-regulated with cell wall associated genes [16]. However, the weak effect of the mutation of the corresponding genes suggests not only that strong redundancies may exist between the transporter genes, but also that alternative routes allowing monolignols to reach the apoplast could exist. The hypothesis of a transport by simple diffusion or diffusion facilitated by channels in the plasma membrane is now also considered [17,18]. This mechanism offers the advantage that it is compatible with the fact that a variety of molecules can be incorporated into the lignin polymer including oligomers formed by combinatorial coupling of monolignol radicals present within the cytoplasm [11]. 

Instead of being transported to the cell wall for polymerization, monolignols can be glycosylated by uridine diphosphate glycosyltransferases (UGT; E.C. 2.4.1.x) on the C4 phenolic hydroxyl group thereby forming *p*-coumaryl alcohol glucoside, coniferin and syringin from *p*-coumaryl-, coniferyl- and sinapyl- alcohols, respectively. In a more general way, most of the intermediates of the lignin biosynthetic pathway including acids, aldehydes and alcohols can also be glycosylated by UGTs. In *Arabidopsis thaliana*, these activities were associated to the UGT72B and UGT72E families [19]

The possibility that monolignol glucosides may represent a storage form of precursors for subsequent lignification remains a matter of debate since coniferin and syringin and to a lesser extent *p*-coumaryl alcohol glucoside, were detected in high amounts in the cambial sap of differentiating xylem of *Picea excelsa* [20]. Since then, coniferin has been detected during the growth period in a number of gymnosperm genus including *Picea*, *Pinus*, *Chamaecyparis*, *Cryptomeria,* and *Larix* [21,22,23,24,25]. The synchronized timing of coniferin production and lignin synthesis in the differentiating xylem could suggest that this glucoside may—in some cases—be a lignin precursor. In contrast to gymnosperms, the amount of coniferin in the differentiating xylem of angiosperms is very limited. A comparative study of monolignol glucosides in *Syringa vulgaris* and *Larix leptolepis* revealed important differences in tissue distribution, patterns and amounts, suggesting that these molecules may play different physiological roles in both angiosperms and gymnosperms [21]. Similarly, a comparison between Japanese cypress (*Chamaecyparis obtusa*) and a hybrid poplar (*Populus sieboldii* X *P. grandidentata*) also highlighted higher amounts of coniferin in the gymnosperm [24]. In a recent study, metabolic profiling of *Arabidopsis thaliana* leaf vacuoles revealed the presence of glycosylated mono-/oligolignols in this compartment [11]. In this latter case, it is difficult to make a link between leaf storage and lignification although whether these molecules can be further transported through the phloem towards other parts of the plant remains an open question. 

When considering these different data, the regulatory role of the glycosylation on the availability of lignin precursors and more generally on the lignification process still remains unclear and deserves further investigation. Indeed, UGTs have been mentioned as actors of lignification [26,27] but to date, there are only very few experimental data supporting this hypothesis. In tobacco, the ectopic expression of a *Populus tomentosa UGT* gene controlled by the *CaMV35S* promoter increased the amounts of Klason lignin by approximately 60% [28]. This unexpected result showed that UGTs may have indirect effect on lignin deposition. In more recent studies, the *Arabidopsis UGT72B1* gene was knocked-out, leading to an ectopic lignification phenotype [29] that was more consistent with the hypothesis of a role played by UGTs in the aglycone lignin precursor homeostasis.

In this work, we investigated the role of the 3 *UGT72E* family members in the regulation of lignification in *Arabidopsis thaliana*. We first showed that the expression of *UGT72E3* is independent of that of the 2 others and that the dynamics of lignin deposition in the corresponding mutant was modified compared to the WT. This was assessed by Raman microspectroscopy and a histological ratiometric approach. We then evaluated the cell wall capacity to integrate monolignols with a bioorthogonal reporter incorporation technique and clearly showed that it was increased in the *ugt72e3* mutant and associated with peroxidase and laccase gene upregulation.

## 2. Results

### 2.1. Impact of UGT72E Mutations on Plant Growth

**Figure 1:**
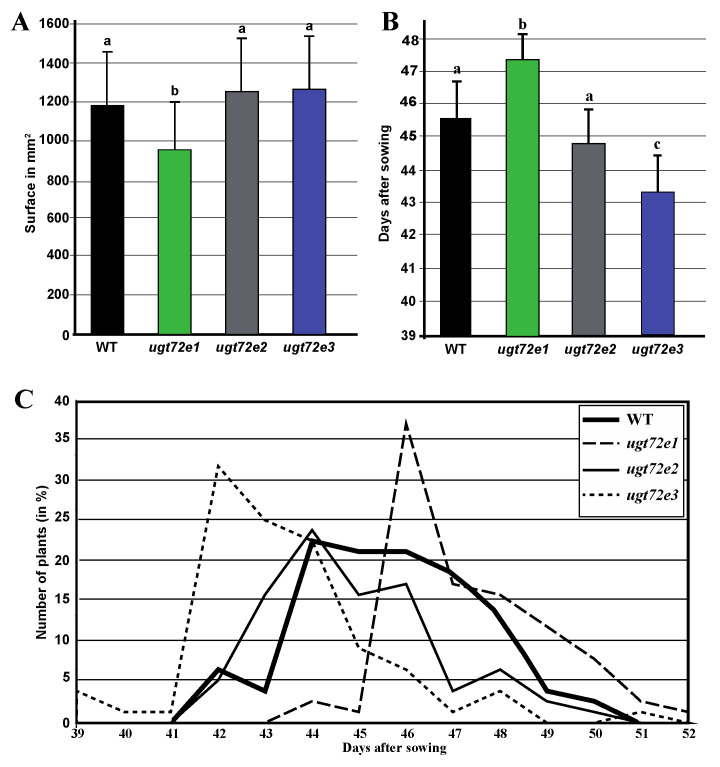
Effect of *UGT72E* gene mutation on the development of *Arabidopsis thaliana*. (**A**). Rosette surface sizes after 28 days of growth. (**B**). Average age of the plants when the flowering stem reached stage 5.1. (**C**): Distribution curve showing the ages of plants within the different genotypes at the flowering stem stage 5.1. Means (+ SD) with the same lowercase letters are not significantly different according to Anova completed with Tukey post-hoc test (*p*-value < 0.01; *n* > 70).

To obtain information about the biological role of the different *UGT72E* genes, the corresponding insertion mutants were investigated for modified phenotypic characters. Although no marked phenotype was visible, specific measurements of the rosette surface of 28-day-old plants (corresponding to the 3.20–3.50 stage [30]) revealed a slight but significant reduction in rosette area for *ugt72e1*, but not for *ugt72e2* and *ugt72e3* mutants when compared to WT plants (Figure 1A) suggesting a possible negative effect of the *ugt72e1* mutation on vegetative growth. Phenotypic differences were also observed at stage 5.1 corresponding to the emergence of the floral stem (Figure 1B). The number of days necessary to reach this stage was significantly increased in the *ugt72e1* mutants, unchanged in *ugt72e2* and decreased in *ugt72e3* compared to WT plants. These differences were also reflected in the distribution of floral stem appearance times observed for the majority of plants within compared genotypes (Figure 1C). Both WT and *ugt72e2* populations show a progressive flowering profile with a maximum at day 44 whereas the two other mutants exhibit a narrower flowering peak occurring either prior to WT (*ugt72e3*) or after (*ugt72e1*), followed by a rapid decline. Taken together these results would suggest that the knock-out of the *UGT72E1* and *UGT72E3* genes have a discrete but significant effect on plant development.

### 2.2. Expression of the UGT72E Genes in the WT and Mutant Floral Stems 

**Figure 2:**
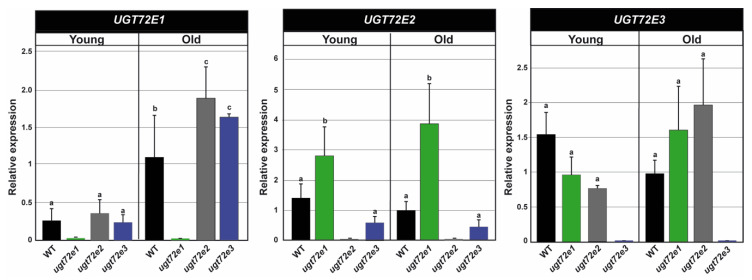
Expression analyses of *UGT72E* genes in wild type (WT) and *ugt72e* mutants. RT-qPCR was used to measure the expression of the genes in the old and young parts of the stem. The values are expressed relative to the WT old stem fragments for each analyzed gene. The expression of the genes corresponding to the mutants is null so was not tested statistically. *PP2A* (AT1G13320) and *PEROXIN4* (AT5G25760) were used as internal reference genes. Means with the same lowercase letters are not significantly different according to *Duncan test* (*p*-value < 0.05, *n* = 3).

To better understand the possible involvement of the *UGT72E* genes in floral stem development and to see whether the inactivation of one of the 3 genes may affect the expression of other *UGT72E* family members, expression analyses were performed on the top and bottom regions of this organ corresponding respectively to “young” and “old” tissues (Figure 2). 

In WT floral stem tissues, all three genes were expressed albeit with different profiles. *UGT72E1* was more highly expressed (approx. 4×) in old tissues compared to young tissues while the expression of *UGT72E2* and *UGT72E3* were similar in both stem regions. In the *ugt72e2* and *ugt72e3* mutants, *UGT72E1* transcript accumulation was slightly but significantly increased in the old part, but not the young part of floral stems as compared to WT. In contrast the accumulation of *UGT72E2* transcripts was only significantly increased in the *ugt72e1* mutant (both young and old zones), but not *ugt72e3* as compared to WT. Finally, no significant change in *UGT72E3* transcript accumulation was observed (young and old zones) in *ugt72e1* and *ugt72e2* mutant tissues.

### 2.3. In Situ Analysis Reveals Increased Cell Wall Lignin Content Only in Ugt72e3 Mutant Stems 

**Figure 3:**
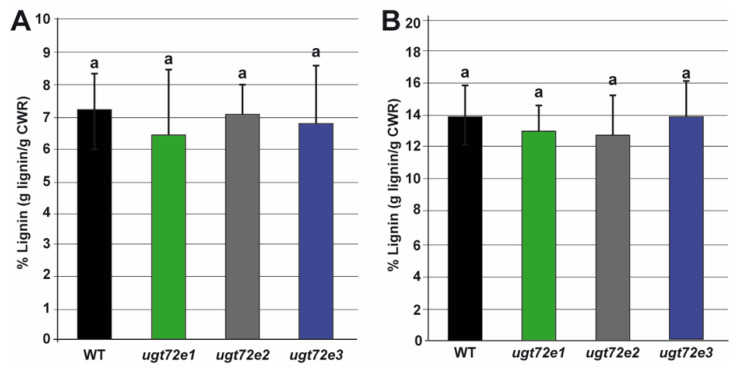
Comparison of floral stem lignin content in WT and *ugt72e* mutants. The lignin dosage was performed by the acetyl bromide method in the young (**A**) parts (*n* = 5 replicates each containing 8 fragments) and old (**B**) parts (*n* = 5 replicates; each containing 4 fragments) of the floral stems. Values are expressed as mean values (±SD). No significant differences were shown by a Kruskal–Wallis test. CWR = cell wall residue.

To see whether the inactivation of different *UGT72E* genes modified lignification, we used a dual approach consisting of (i) a global quantification by acetyl bromide and (ii) an evaluation of relative cellular lignin quantities by a histological ratiometric approach. In both cases, the analyses were performed on the young and old regions of 24 cm-high floral stems. When using the acetyl bromide procedure, no significant differences in lignin contents were observed between the different lines in both parts of the stems (Figure 3). The lignin amounts (g/g CWR) were roughly 2-fold higher in bottom stem fragments compared to the top ones whatever the genotype considered. 

**Figure 4:**
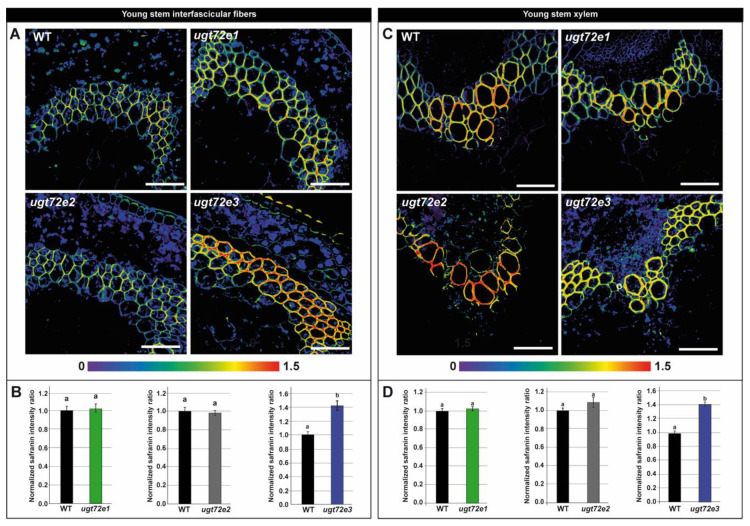
Local lignin content determination on the young parts of the floral stems using safranin O fluorescence-based method on WT and *ugt72e* mutants. The safranin O ratiometric images are shown in (**A**) and (**C**). The colour range used for image representation varies from the non-normalized ratio 0 (purple) to non-normalized ratio 1.5 (red). Scale bar = 50 µm. Quantification was performed on interfascicular fibers (**B**) and xylem (**D**). The normalized safranin intensity ratios (± SD) are represented. Means (± SD) with the same lowercase letters are not significantly different according to Student *t*-test (*p*-value < 0.01, *n* = 3).

**Figure 5:**
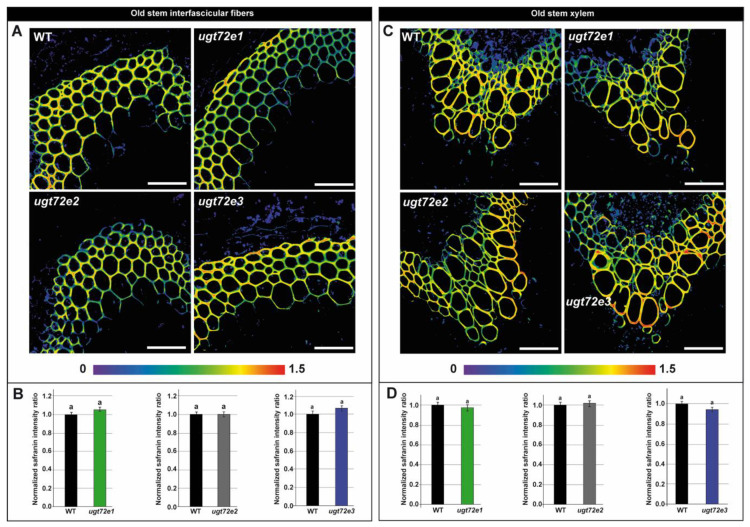
Local lignin content determination on the old parts of the floral stems using safranin O fluorescence-based method on WT and *ugt72e* mutants. The safranin O ratiometric images are shown in (**A**) and (**C**). The color range used for image representation varies from the non-normalized ratio 0 (purple) to non-normalized ratio 1.5 (red). Scale bar = 50 µm. Quantification was performed on interfascicular fibers (**B**) and xylem (**D**). The normalized safranin intensity ratios (± SD) are represented. Means (± SD) with the same lowercase letters are not significantly different according to Student *t*-test (*p*-value < 0.01, *n* = 3).

Since global wet chemistry measurements can mask subtle, but real changes in cell wall lignification, we used a histological approach based on our recent development of a quantitative in situ technique coupling fluorescent microscopy and safranin O staining [31]. There were no significant differences in the lignin content of both interfascicular fibers and xylem from young and old regions of the *ugt72e1* and *ugt72e2* mutant stems compared to the WT (Figure 4 and Figure 5). In contrast, the lignin content of the *ugt72e3* mutant was significantly higher (approx. 40 %) in both tissues from the young part of the stems compared to WT stems. In the old part of the *ugt72e3* stem, the lignin content was similar to the WT.

These results suggest the existence of a functional link between *UGT72E3* gene expression (but not *UGT72E1* or *UGT72E2*) and lignification. To confirm the specificity of the *UGT72E3* gene mutation effect and evaluate a possible functional redundancy with *UGT72E1* and *UGT72E2*, lignin was quantified in the double *ugt72e1,2* mutant used as a control. Indeed, mutant complementation is often used to gain knowledge on the role of a particular gene, but has many disadvantages such as position effect of the T-DNA [32] carrying the non-mutated gene along with the variability of transgene expression by RNA sensing mechanisms [33] but also possible influences of environmental parameters [34]. 

We first showed that the expression level of *UGT72E3* was the same as in the WT (Figure S1). Concerning lignin dosage, our results revealed no significant differences in floral stem lignin content between WT and *ugt72e1,2* samples when evaluated by either the acetyl bromide method (Figure S2) or the safranin O technique (Figure S3). Taken together, these data demonstrate that the observed modifications in cell wall lignin content are most likely caused by *UGT72E3* gene knock-out.

### 2.4. Raman Microspectroscopy Reveals Modifications in Ugt72e3 Cell Walls 

To validate the results obtained by the safranin O methodology and to further explore effects of *UGT72E3* knock-out on cell wall phenotype we used Raman microspectroscopy. 

**Figure 6:**
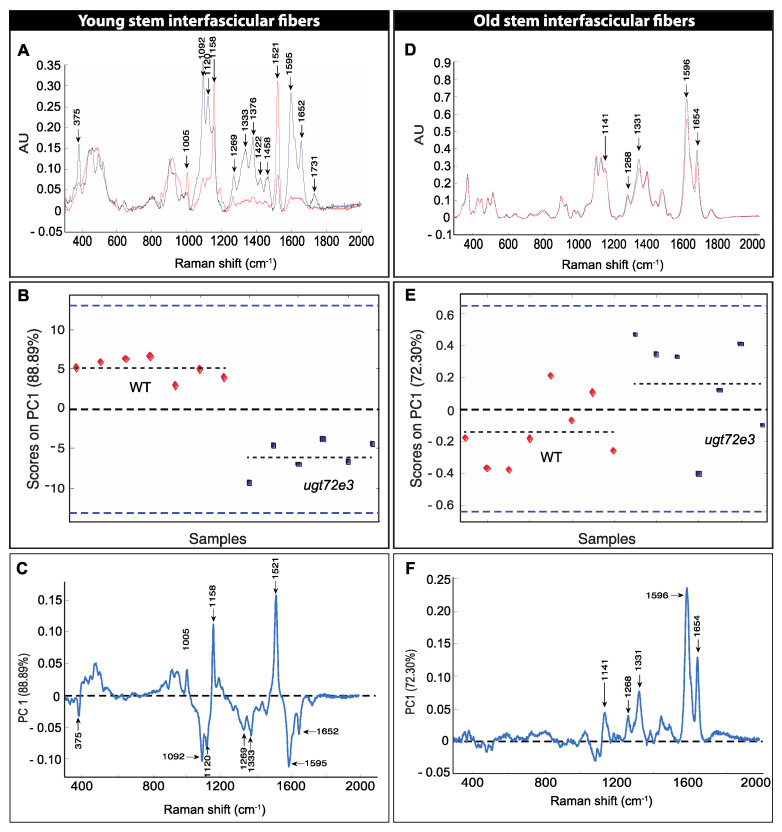
Raman microspectroscopy and principal component analyses (PCA) of WT and *ugt72e3* interfascicular fibers. The results are shown for the young (**A**–**C**) and old (**D**–**F**) parts of the floral stem. (**A**,**D**): Baseline corrected average spectra from 300 cm^−1^ to 2000 cm^−1^. The red spectrum corresponds to the WT and the blue to *ugt72e3*. Arrows indicate major peak Raman shift wavenumbers. (**B**,**E**): Principal component one (PC1) scores of individual WT (red) and *ugt72e3* (blue) spectra following PCA. Each point represented corresponds to an independent individual. (**C**,**F**): Loading vectors for PC1 indicating peaks contributing to the corresponding PC1 scores.

We firstly acquired spectra of interfascicular fiber cell walls from floral stem sections prepared from WT and *ugt72e3* genotypes and generated mean spectra for analysis (Figure S4). Spectra obtained from xylem cell walls were found to be highly variable due to the heterogeneity of the cell types (e.g., vessels, fibers, parenchyma) and sizes within this tissue and it was therefore impossible to generate suitable mean spectra for meaningful comparison of WT and *ugt72e3* xylem cell walls within the context of this study. The comparison of WT and *ugt72e3* baseline-corrected spectra of interfascicular fiber cell walls from young stem regions (Figure 6A) revealed important differences between the two spectra. Four lignin-associated peaks (1269 cm^−1^, 1333 cm^−1^, 1595 cm^−1^, and 1652 cm^−1^) were higher in the *ugt72e3* spectra compared to WT. Similar results were also obtained for major peaks that could be assigned to cellulose (375 cm^−1^, 1092 cm^−1^, and 1120 cm^−1^). In contrast only slight/no differences were observed for peaks related to other major cell wall polymers such as xylans (432 cm^−1^, 454 cm^−1^, 489 cm^−1^, and 516 cm^−1^). In WT spectra 3 peaks (1005 cm^−1^, 1158 cm^−1^, and 1521 cm^−1^) that were assigned to carotenoids and/or xanthophyll pigments [35] were more intense compared to the mutant spectra. 

In order to confirm that the observed differences were statistically relevant we performed principal component analysis (PCA) on the spectra corresponding to the different WT and mutant samples analyzed. Our results show that the first principal component (PC1) represented 88.89% of the total differences observed (Figure 6B). Analyzing scores on PC1 showed that all WT and mutant samples were well discriminated on this component. Examination of the PC1 loading vector (Figure 6C) indicated that the peaks making positive contributions (1005 cm^−1^, 1158 cm^−1^, and 1521 cm^−1^) correspond to pigments while peaks making negative contributions correspond to lignin (1269 cm^−1^, 1333 cm^−1^, 1595 cm^−1^, and 1652 cm^−1^) and cellulose (375 cm^−1^, 1092 cm^−1^, and 1120 cm^−1^) in agreement with the average spectra (Figure 6A). The detection of carotenoids during our analyses is probably due to our technical procedure during which the young tissues containing high amounts of photosynthetic pigments were only fixed during a short period in ethanol. As a consequence, some cellular contents, including pigments, probably interacted with the cell wall material after cutting the tissues with the vibratome. The differences in the degree of contamination between the WT and the *ugt72e3* mutant may possibly be related to a general modification of the cell wall architecture.

The mean spectra (Figure 6D) obtained from old WT and *ugt72e3* floral stem regions were much more similar than corresponding spectra obtained from younger samples (Figure 6A). Nevertheless, when analyzing scores of different samples on the PC1 that accounted for 72.3% of the total variability (Figure 6E), we observed that the WT and *ugt72e3* mutant could still be discriminated (but less so than in younger samples). Examination of the corresponding PC1 loading vector (Figure 6F) indicated that peaks making positive contributions could be assigned to lignin (1141 cm^−1^, 1268 cm^−1^, 1331 cm^−1^, 1596 cm^−1^, and 1654 cm^−1^). Altogether the data obtained by Raman microspectroscopy are in agreement with the results obtained using the safranin O approach. Both methods indicate that cell wall lignin content is significantly increased in the *ugt72e3* mutant compared to WT and that the difference is greater in samples from young floral stem regions. 

### 2.5. Monolignol Reporter Incorporation is Increased in Ugt72e3 Stems

In the light of the observed local increases in *ugt72e3* cell wall lignin content revealed by both the safranin O approach and Raman spectroscopy, we compared the capacity of the WT and *ugt72e3* mutant to incorporate monolignol reporters into the growing lignin polymer in situ using a recently developed bioorthogonal triple labelling strategy [36]. This technology gives detailed information about the potential of the cell wall machinery to incorporate tag-bearing monolignol surrogates (chemical reporters) and therefore provides dynamic information about the lignification process in cell walls. 

**Figure 7:**
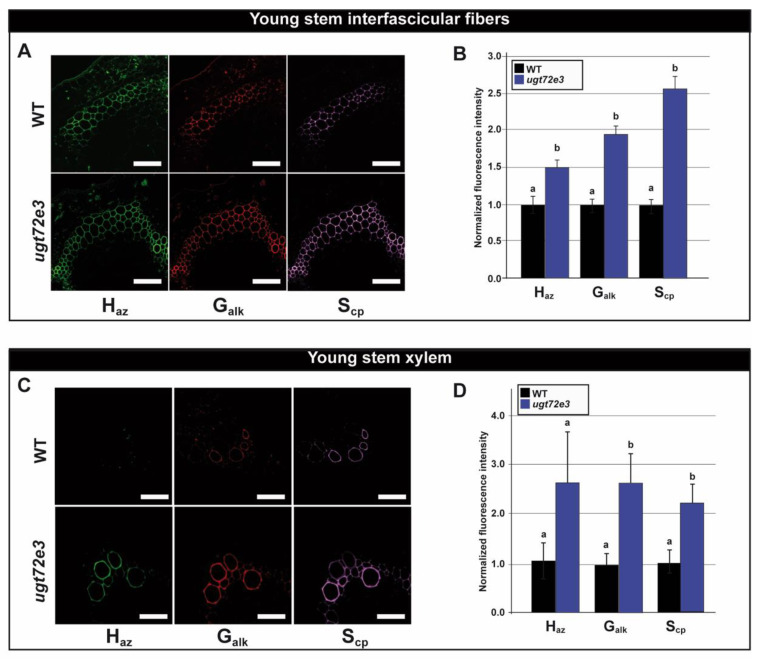
Monolignol incorporation capacity in the young part of the floral stem. The capacity of cell walls to incorporate monolignols into cell walls was evaluated by measuring the signal of fluorescent probes bound to monolignol chemical reporters metabolically incorporated into the lignin polymer. The tagged monolignol associated incorporation signal was localized in the interfascicular fibers and xylem cell walls (**A**,**C**). H_az_ tagged lignin reporter unit signal appears in green, G_alk_ in red and S_cp_ in purple. Scale bar = 50 µm. The associated incorporation signal was quantified in *ugt72e3* and compared to the WT (**B**,**D**). Means (± SD) with the same lowercase letters are not significantly different according to Student *t*-test (*p*-value < 0.01, *n* = 3).

**Figure 8:**
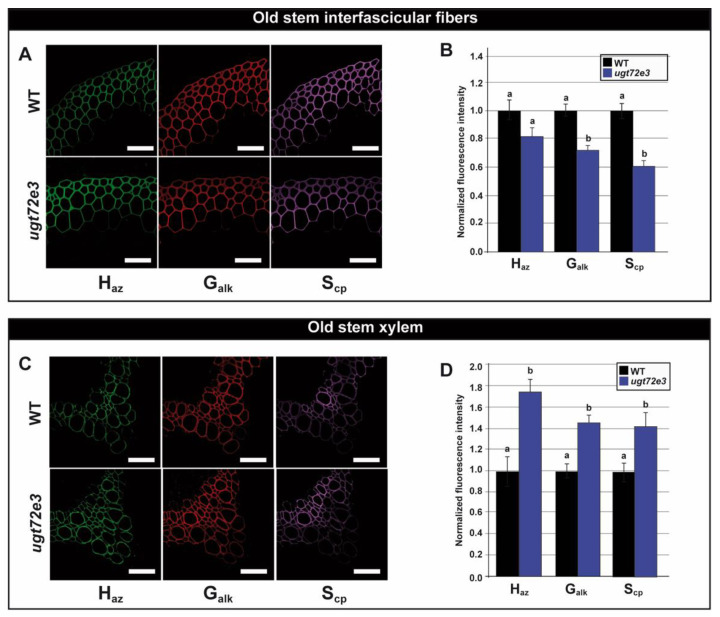
Monolignol incorporation capacity in the old part of the floral stem. The capacity of cell walls to incorporate monolignols into cell walls was evaluated by measuring the signal of fluorescent probes bound to monolignol chemical reporters metabolically incorporated into the lignin polymer. The tagged monolignol associated incorporation signal was localized in the interfascicular fibers and xylem cell walls (**A**,**C**). H_az_ tagged lignin reporter unit signal appears in green, G_alk_ in red and S_cp_ in purple. Scale bar = 50 µm. The associated incorporation signal was quantified in *ugt72e3* and compared to the WT (**B**,**D**). Means (± SD) with the same lowercase letters are not significantly different according to Student *t*-test (*p*-value < 0.01, *n* = 3).

The use of three different chemical reporters H_az_, G_alk_ and S_cp_ (Figure S5) and independent bioorthogonal reactions allowed us to specifically evaluate the incorporation of lignin units corresponding to *p*-coumaryl alcohol, coniferyl alcohol, and sinapyl alcohol molecules respectively. The results are presented as normalized fluorescence intensities extracted from confocal microscopy images for the young (Figure 7) and old (Figure 8) regions of WT and *ugt72e3* mutant floral stems.

When compared to the WT samples, the interfascicular fiber cell walls from young stem regions of the *ugt72e3* mutant incorporated significantly higher amounts of all three reporters (Figure 7A,B) suggesting that the lignification process was modified in this mutant. Examination of xylem from the same region also revealed changes in the lignification process (Figure 7C,D). Here, mutant cell walls incorporated significantly higher amounts of G_alk_- and S_cp_- reporters compared to WT samples. Only the incorporation of H_az_ was not significantly different between WT and mutant xylem cell walls.

In older parts of the stem, reporter incorporation into interfascicular fiber cell walls was altered in *ugt72e3* samples indicating that the lignification process was also modified (Figure 8A,B). However, in contrast to the pattern observed in the young part, here significantly lower amounts of G_alk_- and S_cp_-reporters (but not H_az_-reporters) were incorporated compared to WT samples. Monolignol reporter incorporation was also significantly increased for H_az_-, G_alk_-, and S_cp_-reporters in *ugt72e3* xylem cell walls in comparison with the WT (Figure 8C,D). Taken together, these results clearly suggest that the knock-out of the *UGT72E3* gene induces modifications in the lignification process. 

### 2.6. Ugt72e3 Mutation has no Influence on the Expression of the UGT72B Genes 

**Figure 9:**
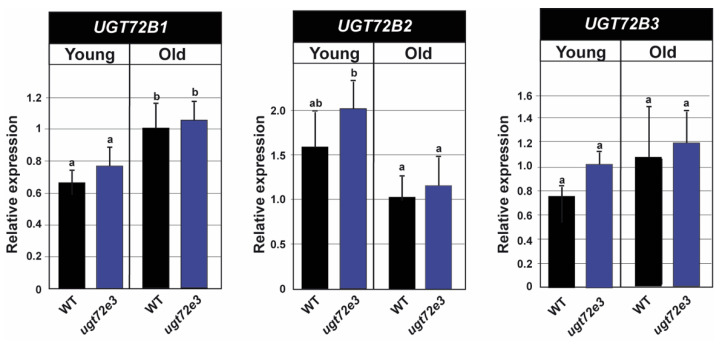
Expression analyses of *UGT72B* genes in WT and *ugt72e3*. RT-qPCR was used to measure the expression of the genes in the old and young parts of the stem. The values are expressed relative to the WT old stem fragments for each analyzed gene. *PP2A* (AT1G13320) and *PEROXIN4* (AT5G25760) were used as internal reference genes. Means (± SD) with the same lowercase letters are not significantly different according to Duncan test (*p*-value < 0.05, *n* = 3).

Our results obtained by three independent methodological approaches (safranin O, Raman, reporter chemistry) all indicated changes to cell wall phenotype in the *ugt72e3* mutant suggesting that *UGT72E3* plays a role in cell wall metabolism. However, it was recently shown that glycosyltransferases from the UGT72B subfamily shared common biochemical functions with *UGT72E2* in *Arabidopsis thaliana* [29]. Like the *UGT72E* genes, *UGT72B1* is expressed in stems and its knock-out modified the expression of not only *UGT72B3* but also *UGT72E2*, suggesting an interconnection between the *UGT72E* and *UGT72B* subfamilies. In order to rule out the possibility that the observed *ugt72e3* phenotype was due to an indirect effect via the *UGT72B* genes, we investigated the effect of knocking-out the *UGT72E3* gene on their expression in floral stems. As shown in Figure 9, the transcript accumulation of all three *UGT72B* genes is not significantly modified in the *ugt72e3* mutant thereby demonstrating that the observed phenotype is not caused by an indirect effect on other lignin-related *UGT72* genes. 

### 2.7. The Expression of PRX and LAC Genes Is Up-Regulated in Ugt72e3 Mutants 

Results obtained with safranin O, Raman and chemical reporter approaches all indicated modified lignification profiles in the *ugt72e3* mutant compared to WT plants. In particular, the chemical reporter strategy suggested that mutants incorporated tagged monolignols to higher levels in the young stem region. Since the formation and extension of the lignin polymer depends mainly on the activities of class III PRX and LAC enzymes we performed RT-qPCR analyses of selected lignin-related redox genes in *ugt72e3* and WT samples. *PRX25* and *PRX71* genes were chosen because their knock-out mutants are characterized by a decrease in total lignins, thinner cell walls, and/or altered lignin structures in stems [37,38]. *PRX64* was also selected since it showed a specific expression in the stems [39] and the corresponding protein was localized in the middle lamella and cell corners of cell walls [40]. We also selected *LAC4* and *LAC17* that have been proposed as key actors in stem lignification [41]. 

**Figure 10:**
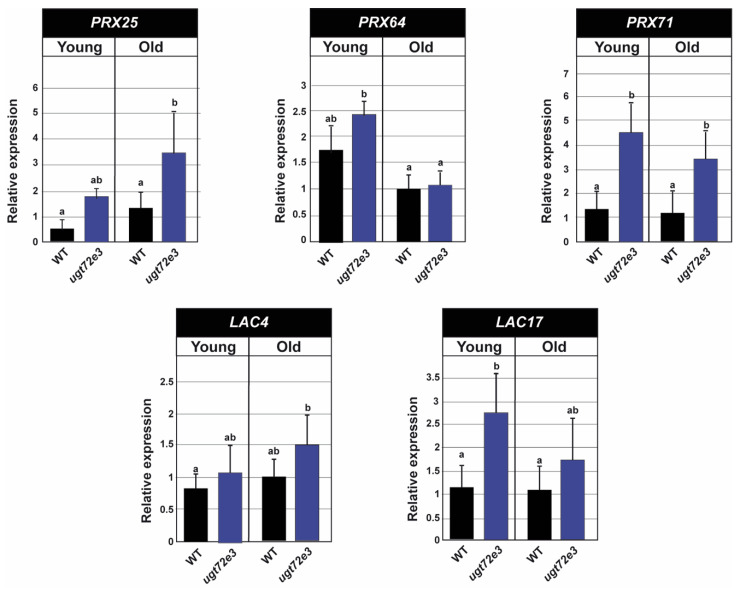
Expression analyses of selected laccases and peroxidases in WT and *ugt72e3*. *PRX25*, *PRX64, PRX71, LAC4,* and *LAC17*, genes were tested by RT-qPCR to measure the expression of the genes in the old and young parts of the stem. The values were relative to the WT old stem fragments for each analyzed gene. *PP2A* (AT1G13320) and *PEROXIN4* (AT5G25760) were used as internal reference genes. Means (± SD) with the same lowercase letters are not significantly different according to *Duncan test* (*p*-value < 0.05, *n* = 3).

In the WT, no differences in the accumulation of the selected *PRX* and *LAC* transcripts were revealed when comparing young and old stem regions (Figure 10). When comparing WT and *ugt72e3* mutant plants in older stem regions, *PRX25* and *PRX71* showed significant increases in transcript accumulation whereas in young stem samples, the accumulation was higher for *PRX71* and *LAC17.* These data clearly indicate that the expression of several key lignin-related redox genes is up-regulated in the floral stems of *ugt72e3* mutants and that the genes possibly involved in monolignol activation are different depending on the age of the stem portion.

## 3. Discussion

Glycosyl-transferase (GT) mediated reactions are considered as among the most important biotransformations on Earth [42]. To date, 110 different GT families are referenced in the CAZy database among which are the UGTs (uridine diphosphate glucosyltransferases) belonging to the GT1 family. There is an important mismatch between the number of glycosylated specialized metabolites in plants, potentially more than 100,000 [43], and the limited number of UGT genes, meaning that the corresponding enzymes can very certainly act on many different substrates. In *Arabidopsis thaliana*, this high functional diversity is reflected by the existence of 21 gene families based on sequence homology (UGT71-92 but not 77). The phylogenetic analysis of the 123 UGT protein sequences showed that they could be divided in 14 groups (A to N) [44]. Since many of the corresponding proteins are still insufficiently characterized, it remains difficult to know whether each group reflects common functions. 

Over the last two decades UGT72E and, more recently UGT72B proteins belonging to the group E have received attention because of their possible role in regulating not only phenylpropanoid metabolism, but also deposition of the important cell wall polymer lignin. In terms of biochemical functions, in vitro tests of enzymatic activities indicate that both UGT72E1 and UGT72E2 can transfer a glucosyl group onto coniferaldehyde and sinapaldehyde and that UGT72E2 and UGT72E3 can transform sinapic acid but also sinapyl and coniferyl alcohols [19]. Ferulic acid can also be glycosylated by UGT72E2. The characterization of the UGT72B1 protein also showed that while the enzymatic activity was redundant with that of UGT72E2 [29], the Km value was higher in the former.

While a recent study [29] demonstrated that UGT72B1 is essential for normal cell wall lignification in *Arabidopsis*, the situation is less clear for members of the UGT72E family. Analyses of mutants over-/under-expressing *UGT72E1/2/3* genes indicated changes in the levels of monolignol glucosides however, no significant effect on lignin deposition was observed [45]. In this paper we decided to reevaluate the potential involvement of the UGT72E family in lignification using chemical quantification together with several powerful in situ imaging approaches to characterize the *Arabidopsis ugt72e1*, *ugt72e2,* and *ugt72e3* mutants.

Lignin contents were estimated using acetyl bromide. This dosage protocol was first developed for small wood samples [46] and later adapted for herbaceous species. It is particularly appropriate for lignin estimation in the floral stem of *Arabidopsis* because of the low amounts of cell wall xylans, which can potentially interfere with the spectrometric absorption [47] when using perchloric acid during the lignin extraction. In our protocol, the absence of artifactual furans [48] was also shown by checking the absence of a shift in the absorption spectrum to wavelengths lower than 280 nm. In our hands, this approach did not reveal any differences in lignin content between the *ugt72e* mutants and wild type. Chemical estimations of lignin, however, are generally destructive, providing an average value and resulting in the loss of detailed spatial information about cell wall lignin levels [49]. We therefore decided to use complementary in situ imaging techniques to investigate whether *UGT72E* gene knock-out affected cell wall lignin.

We firstly implemented a new quantification approach that we have developed based upon the potential of fluorescent microscopy coupled to safranin O staining [31]. Using this technique, it is now possible to generate high-definition spatial information about lignin localization as well as quantitative information based on ratiometric emission measurements. Our results showed that lignin deposition was significantly changed only in the *ugt72e3* mutant, but not in *ugt72e1* or *ugt72e2*. Lignin levels in both the xylem and interfascicular fibers from the younger part of *ugt72e3* stems were higher when compared to the WT. In contrast, no statistical differences were observed in the bottom part of the organ that contains more mature cells.

It seems therefore that the knock-out of the *UGT72E3* gene accelerates cell wall lignification in younger tissues but does not influence the final amounts at the end of cell wall development. In order to confirm this observation we then used Raman microspectroscopy and a chemical reporter strategy [49]. Both of these approaches confirmed that cell wall lignin levels were significantly increased in *ugt72e3* mutants compared to WT plants and provided strong evidence for the idea that the *UGT72E3* gene is involved in the regulation of cell wall lignification. Expression analyses showed that the accumulation of transcripts for the 3 *UGT72B* family members were not significantly modified in the mutant thereby ruling out the possibility that the phenotype was due to an indirect effect on the *UGT72B* genes.

The existence of a possible relationship between UGTs and lignification has been often mentioned but is only poorly supported by experimental data [13,26,27,50]. In tobacco plants, the ectopic expression of the *Populus tomentosa PtGT1*, homologous to the 3 *UGT72E Arabidopsis* genes, led to the accumulation of higher lignin levels in the xylem [28]. The interpretation of this result is not really intuitive when considering that UGTs may reduce the flux of monolignols towards the cell wall. It is possible that the use of the strong *CaMV35S* promoter to control UGT gene expression led to significant disruption of gene function in this case. It should also be taken in account that no enzymatic activity towards monolignols was associated with the corresponding recombinant protein, suggesting that the impact of this UGT on lignin biosynthesis is not directly through the glycosylation of monolignols, In *Arabidopsis*, the *ugt72b1* mutant was characterized by ectopic lignification in areas around pith tissues and interfascicular fibers [29]. The increase in the quantities of lignins in the cell walls does agree with the fact that this UGT could catalyze the glucose conjugation of monolignols but still, the very strong impact of the mutation is surprising for this gene. 

The ectopic expression of *PtGT1* in tobacco resulted in an early flowering phenotype [28]. In our work, the development analysis of the *ugt72e3* mutant also showed that it had an advanced flowering phenotype. This relationship between the development of the floral stem and its lignin contents is difficult to explain. In different plant species, the flowering time has often been linked to lignification when searching for a marker of the biomass yield. In *Medicago truncatula*, a delayed flowering mutant had higher biomass and lower lignin amounts compared to the wild type at the same age [51]. Nevertheless, the correlation between flowering time and lignification may dependent on the clade. In maize, the brown midrib *bm1* heterozygote lines flowered significantly earlier than the wild-type plants but have a reduced content and altered subunit composition lignin polymer [52]. It is possible that the anatomical differences including the absence of secondary tissues in monocots may explain the observed difference with the dicots.

In the light of our results it therefore seems that the mutation of the *UGT72E3* gene impacts lignin deposition, showing that glycosylation could act as a regulator of the monolignol flux. Indeed, when considering the recent suggestion that monolignols cross the plasma membrane by passive diffusion, it is likely that the decreased lipophilic nature of monolignol glycosides resulting from glycosylation would reduce monolignol flow to the cell wall [17]. The role of glycosylation was also highlighted during a study on the potential role of ABC transporters [53] suggesting that the glycoconjugates are transported into the vacuoles and aglycones go towards the cell wall through the plasma membrane. If the *UGT72E* gene is indeed involved in regulating monolignol flux to the cell wall then one would expect there to be an inverse relationship between lignin levels and amounts of glycosylated monolignols. Based on a recent metabolomic study on the inner seed coats of pomegranate [54], it was proposed that the high amounts of monolignols accumulated in the inner seed coats could either be transported to the cell wall and used as lignin precursors leading to a hard-inner seed coat phenotype, or else conjugated into monolignol glucosides and stored, causing a soft-seeded cultivar. In flax, the same inverse correlation was described in a comparative study of the amounts of glysosylated phenolic compounds in outer stem tissues containing fibres with very low amounts of cell wall lignins, and the lignin-rich xylem tissues [55]. In this case, phenolic glycoside levels were higher in the lignin-poor outer tissues compared to the lignin-rich inner tissues. In this same species, a transcriptomic approach showed that the expression of a *UGT* gene close to the *UGT72E* family, was downregulated in the highly lignified bast fibre *lbf* mutant [50] further supporting the idea of this relationship between the lignification status and monolignol glycosylation activity.

Given the hypothesis that the knock-out of the *UGT72E3* gene leads to increased monolignol flow to the cell wall also raises the question of the capacity of cell walls to incorporate these precursors. Before monolignols can be incorporated into the growing lignin polymer, they must first be oxidized by cell wall located peroxidases and/or laccases. If these enzymes are not working close to their maximum efficiency then increasing monolignol flow to the cell wall should lead to increased incorporation and higher cell wall lignin levels. On the other hand, if lignin redox enzymes are biochemically saturated then increasing the monolignol flow will not change lignin levels. To gain insight into this question we used a novel bioorthogonal triple labelling strategy based on the use of chemical reporters to compare lignification dynamics in the floral stem of *ugt72e3* mutants and WT plants [36]. In addition, we also determined expression levels of selected lignin-related peroxidase and laccase genes.

Our results showing that *UGT72E3* knock-out stimulated the capacity of mutant cell walls to incorporate monolignol chemical reporters in interfascicular fibre and xylem cell walls of young stem regions is coherent with, not only the data obtained by the safranin O ratiometric approach and Raman microspectroscopy, but also the hypothesis of increased monolignol flow to the cell wall. The observation that the accumulation of *PRX71* and *LAC17* transcripts also increased in this stem region would suggest that these genes are up-regulated in response to the increased monolignol flow to the cell wall resulting from decreased glycosylation. The fact that *PRX25*, *PRX64,* and *LAC4* expression were unchanged could indicate that the corresponding proteins are present in a constitutive way and are not saturated by increased monolignol flow or alternatively they may be less involved in the lignification of cell walls in the younger part of the stem. In tissues from old stem regions, a more complex pattern emerged with decreased lignin G_alk_- and S_cp_- reporter incorporation in *ugt72e3* interfascicular fibre cell walls compared to WT associated with increased incorporation of all 3 reporters in xylem tissue. The observed reduction of G_alk_- and S_cp_- reporter incorporation in interfascicular fibre cell walls is potentially related to the fact that in this region of the stem, the lignin levels in these cell walls are very similar in both WT and mutant plants as demonstrated by the safranin O and Raman microspectroscopy results. This could suggest that the lignification process is winding down in these cells in agreement with reduced reporter incorporation. In contrast, the formation of new cells in the xylem is associated with continued lignification as shown by the increased incorporation of all 3 reporters. The gene expression data for the old stem region showed that *PRX71* and *PRX25*, but not *LAC17*, were both more highly expressed in the *ugt72e3* mutant, most likely representing a developmentally related switch in the nature of the lignin related redox enzymes. The analysis of the corresponding mutants with our in vivo triple labelling strategy could provide more information on the precise role of these different *PRX* and *LAC* genes. Further investigations using a high throughput transcriptomics approach on the *ugt72e3* line will also provide information on the expression of lignin biosynthetic genes and the genes encoding transcription factors known to be involved in the regulation of the phenylpropanoid pathway. It is indeed possible that a higher amount of monolignols were synthesized due to the mutation.

In conclusion, this study has generated several key results supporting the hypothesis that monolignol glycosylation plays a regulatory role in controlling cell wall lignification in the *Arabidopsis* floral stem, especially in the young tissues. The use of three independent high-performance imaging techniques combined with gene expression data provides clear evidence for a role of the UGT72E3 protein in this process.

## 4. Materials and Methods 

### 4.1. Plant Material and Sampling

All the *Arabidopsis thaliana* plants were in the Col-0 background. The three T-DNA insertion mutant lines GK-340H02, SM_3_20654 and SAIL_1279_D02 corresponding respectively to the *UGT72E1* (At3g50740), *UGT72E2* (At5g66690), and *UGT72E3* (At5g26310) genes were ordered from NASC (http://arabidopsis.info) and the homozygotes were selected by PCR. Double mutants *ugt72e1,2* were obtained by crossing *ugt72e1* with *ugt72e2*. In each mutant, the T-DNA was positioned at the 5′ end of the unique exon.

The seeds were stratified during 72 h at 4 °C in the dark. Plants were grown in GroBank chambers (CLF Plant Climatics GmbH, Wertingen, Germany) under 12 h light (100 PAR light intensity) at 22 °C and 12 h dark at 20 °C. After 6 weeks, the photoperiod was changed to 16 h light, 8 h dark. For the phenotyping analyses, the rosette surfaces of at least 70 plants were measured on 28-day-old plants corresponding to the 3.20–3.50 stage [30] using FIJI v2.0 [56]. The age of the plants reaching the 5.1 stage, corresponding to the emergence of the floral stem, was also annotated. For lignin analyses, floral stems were collected when they reached 24 cm. The first bottom centimeter was discarded and a sample including either the above centimeter was used for safranin and biorthogonal labelling analyses or the 8 first centimeters used for lignin dosage by acetyl bromide or gene expression approaches (old stem regions). A second 8 cm fragment was collected for lignin dosage and gene expression analysis above the 11 first centimeters from the bottom of the stem. The central part of this fragment measuring 1 cm was used for safranin and biorthogonal labelling approaches (young stem regions).

### 4.2. Determination of Gene Expression by RT-qPCR

Top (young) and bottom (old) stem fragments from three 6-week-old plants for each biological replicate were pooled, frozen in liquid nitrogen and stored at −80 °C. Total RNA was extracted from ground tissues using Tri-Reagent (MRC, Inc., Dundee, Scotland, UK) according to manufacturer’s instructions, treated with TURBO DNA-free DNase (Thermo Fisher, Courtaboeuf, France) and quantified by Nanodrop. First strand cDNA was obtained from 1µg total RNA using iScript™ cDNA Synthesis Kit (Bio-Rad, Marnes-la-Coquette, France) according to manufacturer’s instructions. PCR was performed on Mx3005P (Agilent, Les Ulis, France) in duplicate on cDNA pooled from two independent reverse transcription reactions on three independent biological replicates. Reaction mixtures containing 10 µL of iQ SYBR Green Supermix (Bio-Rad, Marnes-la-Coquette, France), 375 nM each primer (Table S1) and 5 µL of cDNA diluted 6 times were incubated for 3 min 30 s at 95 °C followed by 40 cycles at 95 °C for 30 s and 60 °C for 30 s. Gene expression was normalized to the Protein Phosphatase 2A subunit A3 gene *PP2A* (AT1G13320) and *PEROXIN4* (AT5G25760) [57]. Relative expression was determined as previously described [58]. One-way analyses of variance were performed with R, then grouping was done with Duncan multiple range test (*p*-value < 0.05).

### 4.3. Cell Wall Residue Preparation and Lignin Quantification 

For each biological repeat, 4 or 8 fragments were collected from the bottom or top parts of the floral stems. Cell wall residues (CWR) were prepared as previously described [59]. The extensive solvent extraction method was chosen to remove proteins. It globally consisted in successive treatments of the plant material with the addition of 80% (*v*/*v*) ethanol, 100% acetone and CHCl_3_/methanol (1:1). The procedure of cell wall isolation also included amylase and amyloglucosidase treatments (the equivalent of 20 and 40 units respectively, per gram of cell wall sample) for starch removal. Finally, cell wall samples were ground in a bead miller (Mixer Mill MM 301; Retsch, Haan, Germany) at 30 Hz for 2 × 3 min of milling with 5 min pauses in between. The lignin quantification of CWR was performed using a modified acetyl bromide procedure [60]. Ten mg of cell wall sample were incubated in 5 mL of 25% (*w*/*w*) acetyl bromide in glacial acetic acid (extemporized preparation) at 70 °C for 30 min to achieve complete dissolution of residues. After cooling on ice, 2 mL of the mixture were incubated for 30 min in 0.5 M NaOH in glacial acetic acid. Lignin content was determined by measuring the OD at 280 nm and using ε = 20 g^−1^·L·cm^−1^

### 4.4. Sample Preparation for Imaging Analyses

Stem fragments were collected as described in 4.1 and embedded in agarose 4% before sectioning with a vibroslicer (VT-1000S, Leica, Wetzlar, Germany) to a thickness of 80 µm. The sections were placed in Murashige and Skoog half-strength (MS1/2) solution when used for biorthogonal labelling chemistry or fixed for 3 h. in 70% ethanol at room temperature under shaking and then kept at 4 °C before safranin O staining and Raman microspectroscopy.

### 4.5. Safranin O Ratiometric Imaging

Safranin O staining and analyses were performed as previously described [31]. Images from 3 independent biological replicates for each genotype and tissue were acquired. A Nikon A1R confocal equipped with a 60×/1.4 aperture oil immersion objective (Plan APO VC) and the NIS Element AR3.0 software was used (Nikon, Tokyo, Japan). Acquisition parameters were the following: track 1 (ex: 488 nm, BP: 525/50), track 2 (ex: 561 nm, BP: 595/50). Images were treated using FIJI v2.0 [56] and analyzed with the associated macro developed for this method as previously described [31]. Lignin appears in colors ranging from purple (low amounts) to red (high amounts).

### 4.6. Raman Microspectroscopy

Confocal Raman hyperspectral analyses were performed with a Labram HR Evolution (Horiba Scientific, Villeneuve-d’Ascq, France) equipped with a two-dimensional EMCCD detector (1600 × 200 pixels sensor), a 50× Fluotar LWD objective (Leica, Wetzlar, Germany) and a 545-nm laser with a power of 45 mW at the sample. The spectral range of 300 to 2000 cm^−1^ was used for acquisitions. Ten accumulations of 1 s with 100% laser power were used.

The spectra were collected with the LabSpec 6 software (Horiba Scientific, Lille, France). A minimum of 100 spectra (interfascicular fibres) and 80 spectra (xylem) per genotype from 6 to 8 plants distributed on 3 independent biological replicates were acquired. For data processing, once acquired, the hyperspectral images were unfolded and the spectra were baseline corrected using the Whittaker filter method (lambda = 10,000, λ= 0.01), normalized on the 590–630 cm^−1^ (top stem) and 375 cm^−1^ (bottom stem) bands and mean-centered with MatLab R2018b software. PCA were applied on the treated spectra of both genotypes from top and bottom stem samples.

### 4.7. Chemical Reporter Imaging

Chemical reporters H_az_, G_alk_ and S_cp_ were synthesized as previously described [36,61,62]. Natural and tagged monolignols were metabolically incorporated in the plant samples at a final concentration of 5 µM for 20 h (Figure S5). Bioorthogonal ligation of a specific fluorescent probe to each incorporated reporters H_az_, G_alk_, and S_cp_ was carried out according to our previously described triple labelling technology with modifications [36], using a combination of inverse electronic demand Diels-Alder chemistry, Copper-catalyzed alkyne-azide cycloaddition and Strain-promoted alkyne-azide cycloaddition. Cyclopropane-reactive Tetrazine-Cy5 (Jena Bioscience, Jena, Germany) and alkyne-reactive Azidefluor 545 (Sigma-Aldrich, Saint-Louis, MO, USA) probes were used at 5 µM to label incorporated S_cp_- and G_alk_-units, respectively. DBCO-PEG_4_-Rhodamine Green (Jena Bioscience, Jena, Germany) was used at 2.5 µM to label incorporated H_az_-units. Confocal microscopy was done with the same equipment used for safranin imaging with the following acquisition parameters: track 1—lignin autofluorescence (ex: 405, BP: 450/50); track 2—H units (ex: 488, BP: 525/50); track 3—G units (ex: 561, BP: 595/50); track 4—S units (ex: 561, BP: 700/75). An additional channel was acquired with transmitted line detector to obtain morphological images. Images from natural or tagged monolignol units from the different plant lines were acquired with the same acquisition parameters on the same day for valid comparison. Pictures were treated using FIJI v2.0 [56]. Quantitative measurements were performed using an in-house developed macro. Image processing consisted in two phases: the threshold background determination and the analysis. Threshold backgrounds were determined for the channels 2, 3, and 4 on samples treated with natural monolignols. A mean background threshold was determined for each sample from 3 images. Images from samples treated with tagged monolignols were converted in 32-bit images and the channels 2, 3, and 4 were extracted. The determined signal background was subtracted for each channel and pixels below the threshold background were classified as “NaN background”. A region of interest was determined for each sample and the protocol was applied on the three obtained images. Finally, signal intensity was measured and comparison between the different plant lines was performed.

## Supplementary Materials

Supplementary materials can be found at https://www.mdpi.com/1422-0067/21/17/6094/s1. Figure S1: Expression analyses of the *UGT72E3* gene in WT and *ugt72e1,2* mutant. Figure S2: Lignin amounts in the WT and the *ugt72e1,2* mutant. Figure S3: Local lignin content determination on the floral stems using safranin O fluorescence-based method on WT and *ugt72e1,2* mutants. Figure S4: Raman microspectroscopy analysis of the interfascicular fibres. Figure S5: Chemical reporters used in this work. Table S1: Primers used for RT-qPCR.
